# Essentials of Hair Care often Neglected: Hair Cleansing

**DOI:** 10.4103/0974-7753.66909

**Published:** 2010

**Authors:** Zoe D Draelos

**Affiliations:** Department of Dermatology, Duke University School of Medicine, Durham, North Carolina, USA

**Keywords:** Essential of hair care, shampoo, conditioner

## Abstract

Why does the selection of hair cleansing products and conditioners seem complex? Why are there clear, opalescent, green, blue, glittery, cheap, expensive, thick, thin, fragrant, and unscented varieties of shampoos and conditioners? Why the whole cleansing process cannot be simplified by using the same bar soap used on the body for the hair? Does the shampoo selected really make a difference? What can a conditioner accomplish?

## INTRODUCTION

A shampoo is technically designed to clean the scalp of sebum and prevent the development of folliculitis and seborrheic dermatitis. Shampoos are intended to rid the hair of sebum, sweat components, desquamated stratum corneum, styling products, and environmental dirt.[1] The idea of beautifying the hair is really a secondary concern that is primarily addressed by a conditioner.

Beautifying the hair is really quite a complex task. The average woman possesses four to eight square meters of hair surface area to clean.[2] Bar soap was used to clean the hair until the mid 1930s, when liquid coconut oils became available, which allowed the formulation of a liquid soap that lathered and rinsed better than a bar soap.[3] At present, bar soaps are not recommended for hair cleansing because they leave behind a soap scum when mixed with hard water that is difficult to rinse from the hair and scalp. This may be one of the aggravating factors for seborrheic dermatitis in some older men who use a bar soap to clean the scalp. Thus, there really is a need for well-formulated shampoos that both clean and beautify the hair.

## BASIC SHAMPOO FORMULATION

Shampoos are basically liquid cleansers based on synthetic detergents blended to achieve the desired amount of cleansing given the condition of the hair. Some ingredients are added for actual hair and scalp cleansing, while others are added to impart desirable aesthetic characteristics to the shampoo. The basic recipe for a shampoo is listed in Table 1.[4]

**Table 1:** Basic shampoo ingredient formulation and functions

Detergents	Functions to remove environment dirt, styling products, sebum, and skin scales from the hair and scalp
Foaming agents	This agent allows the shampoo to form suds, as consumers equate cleansing with foaming even though the two are unrelated
Conditioners	Leave the hair soft and smooth after sebum removal by the detergent
Thickeners	Thicken the shampoo, as consumers feel that a thick shampoo works better than a thin shampoo
Opacifiers	Added to make a shampoo opaque as opposed to translucent for aesthetic purposes, unrelated to cleansing
Sequestering agents	Functions to prevent soap scum from forming on the hair and scalp in the presence of hard water; The basic difference between a liquid shampoo and a bar cleanser
Fragrances	Added to give the shampoo a consumer-acceptable smell
Preservatives	Prevent microbial and fungal contamination of the shampoo before and after opening
Specialty additives	Treatment ingredients or marketing aids added to impart other benefits to the shampoo, besides hair and scalp cleansing

## SHAMPOO DETERGENTS

Shampoos function by employing detergents, also known as surfactants, which are amphiphilic. This means that the detergent molecule possesses both lipophilic, meaning oil attracting, and hydrophilic, meaning water attracting, sites. The lipophilic site binds to the sebum, while the hydrophilic site binds to water, allowing removal of the sebum with water rinsing.[5] Table 2 lists the shampoo detergents currently available to the cosmetic chemist for use in shampoo formulations.[6] Typically, several detergents are combined together to achieve the desired result. For example, if the shampoo is intended for oily hair, detergents with strong sebum removal qualities are selected, while if the shampoo is intended for permanently waved or dyed hair, mild detergents are selected to reduce sebum removal. The art of shampoo formulation is picking the right detergent combination to cleanse the scalp and beautify the hair simultaneously.

**Table 2:** The most common shampoo detergents

Sodium laureth sulfate
Sodium lauryl sulfate
TEA lauryl sulfate
Ammonium laureth sulfate
Ammonium lauryl sulfate
DEA lauryl sulfate
Sodium olefin sulfonate

There are five basic categories of shampoo detergents: anionics, cationics, amphoterics, nonionics, and natural surfactants.[7] Each of these groups possess different hair cleansing and conditioning characteristics. While this may seem somewhat confusing, an understanding of the detergents is the key to determining which shampoo is most appropriate for a patient with a given hair problem.

## ANIONIC DETERGENTS

Anionic detergents are the most popular surfactants used in basic cleansing shampoos in the current market. They are named for their negatively charged hydrophilic polar group. Anionic detergents are derived from fatty alcohols and are exceptionally adept at removing sebum from the scalp and hair. Unfortunately, the aesthetics of thoroughly cleaned hair are not well accepted by the consumer. Hair devoid of all sebum is harsh, rough, subject to static electricity, dull, and hair that needs detangling. The art of shampoo formulation is achieving the right balance between hair that is sufficiently clean and hair that is too clean. There are several common detergents categorized within the anionic group:

### Chemical class:

#### Lauryl sulfates

Most shampoos designed to produce good hair cleansing will contain lauryl sulfate as the second or third indgredient listed on the label, with water being the primary ingredient. The detergent listed first is the primary cleanser in highest concentration and the detergent listed second is the secondary cleanser designed to complement the short comings of the primary detergent. Examples of lauryl sulfate detergents include: sodium lauryl sulfate, triethanolamine lauryl sulfate, and ammonium lauryl sulfate. These ingredients are popular primary cleansers, as they work well in both hard and soft water, produce rich foam, and are easily rinsed. They are excellent cleansers, but hard on the hair requiring careful selection of a secondary detergent and possible use of a conditioning agent as part of the shampoo formulation. Lauryl sulfates are commonly used in shampoos for oily hair.

#### Laureth sulfates

Laureth sulfates are one of the most commonly used primary detergents in general shampoos designed for normal-to-dry hair. They provide excellent cleansing, but leave the hair in good condition. Consumers like these detergents, as they produce abundant foam. Examples of detergents that fall into this chemical class, as listed on the shampoo label are: sodium laureth sulfate, triethanolamine laureth sulfate, and ammonium laureth sulfate.

#### Sarcosines

Sarcosines are generally not used as primary detergents, as they do not remove sebum well from the hair. However, they are excellent conditioners and commonly used as the second or third listed detergent on the shampoo ingredient list. Sarcosines are used in conditioning shampoos and dry hair shampoos. Detergents of this class may be listed on the shampoo label as: lauryl sarcosine and sodium lauryl sarcosinate.

#### Sulfosuccinates

Sulfosuccinates are a class of strong detergents useful in removing sebum from oily hair. For this reason, they are a common secondary surfactant in oily hair shampoos. Examples of ingredients that fit into this class are disodium oleamine sulfosuccinate and sodium dioctyl sulfosuccinate.

## CATIONIC DETERGENTS

Whereas the anionic detergents we have been discussing are named for their negatively charged polar group, the cationic detergents are named for their positively charged polar group. The cationic detergents are not nearly as popular in current shampoos as the anionic detergents because they are limited in their ability to remove sebum and do not produced abundant lather. They cannot be combined with other anionic detergents, which is another drawback. Cationic detergents are primarily used in shampoos where minimal cleansing is desired, such as in daily shampoos designed for permanently dyed or chemically bleached hair. Here minimal sebum removal is desired, but the cationic detergents are excellent at imparting softness and manageability.[2]

## NONIONIC DETERGENTS

The nonionic detergents are the second most popular surfactants, behind the anionic detergents, and bear the name nonionic, as they have no polar group. These are the mildest of all surfactants and are used in combination with ionic surfactants as a secondary cleanser.[8] Examples of nonionic detergents currently used in shampoos are polyoxyethylene fatty alcohols and polyoxyethylene sorbitol esters and alkanolamides.

## AMPHOTERIC DETERGENTS

The term amphoteric refers to substances that have both a negatively charged and a positively charged polar group. Thus, amphoteric detergents contain both an anionic and a cationic group, which allows them to behave as cationic detergents at lower pH values and as anionic detergents at higher pH values. These unique properties make amphoteric detergents quite unique. Within the amphoteric detergent category, there are several subgroups that include the betaines, sultaines, and imidazolinium derivatives. Special uses for amphoteric detergents include ingredients such as cocamidopropyl betaine and sodium lauraminopropionate, which are found in baby shampoos. These detergents are non-irritating to the eyes, accounting for the non-stinging characteristics of baby shampoo. Amphoteric detergents are also used in shampoos for fine and chemically treated hair because they foam moderately well, while leaving the hair manageable.

## NATURAL DETERGENTS

The synthetic detergents previously discussed have largely replaced natural detergents, until recently, when botanically based hair care products have made a resurgence. Natural surfactants come from plants such as sarsaparilla, soapwort, soap bark, and ivy agave. These natural saponins have excellent lathering capabilities, but are poor cleansers thus must be present at high concentration. Usually, they are combined with other synthetic detergents that have been outlined earlier.[5] The synthetic detergents provide most of the hair and scalp cleansing, while the botanicals are largely added for marketing purposes.

## SHAMPOO FORMULATION

All shampoo formulations contain the same basic ingredients. The variety of shampoos in the marketplace might be rather confusing, but the ingredient categories are standard to a large extent. The categories of shampoo ingredients are discussed next.

## FOAMING AGENTS

One of the most important attributes of a shampoo from a consumer perspective is the foaming ability. Consumers are convinced that a shampoo that foams poorly also cleans poorly. This is not the case. Most shampoos contain foaming agents to introduce gas bubbles into the water. The foam, also known as lather, is important, as it functions to spread the detergent over the hair and scalp, but it does not participate in cleaning. It is true that a shampoo applied to dirty hair will not foam as much as the same shampoo applied to clean hair. This is due to the sebum inhibiting bubble formation. Thus, a shampoo will foam less on the first shampooing and more on the second shampooing. Some of the prescription corticosteroid shampoos do not foam as much as cosmetic shampoos, but this does not mean their cleaning is inadequate.

## THICKENERS AND OPACIFIERS

These ingredients are used to change the physical and optical properties of the shampoo. Thickeners increase the product viscosity, which many consumers feel makes a better shampoo. Opacifiers are used to make shampoos have a pearly shine, which offers no improved cleansing, only an optical effect.

## SEQUESTERING AGENTS

Another important shampoo ingredient that does not participate in cleansing is the sequestering agent. The function of sequestering agents is to chelate magnesium and calcium ions preventing the formation of insoluble soaps, known as ‘scum.’ Without sequestering agents, shampoos would leave a scum film on the hair making it appear dull. This same film can also form on the scalp contributing to itching and ultimately some of the symptoms of seborrheic dermatitis. For this reason, patients should be encouraged to use shampoo and not bar soap when cleansing the hair.

## CONDITIONERS IN SHAMPOO FORMULATIONS

Even as the main intent of a shampoo is to cleanse the scalp and hair, over cleansed hair is not cosmetically acceptable. Hair that is completely devoid of sebum is harsh, difficult to style, and dull. Some persons wish to shampoo daily as a hygiene ritual, whether there has been adequate sebum production or not. Thus, shampoos formulated for dry, damaged, or chemically treated hair frequently contain a conditioner. The conditioner functions to impart manageability, gloss, and antistatic properties to the hair. These are usually fatty alcohols, fatty esters, vegetable oils, mineral oils, or humectants. Commonly used conditioning substances include hydrolyzed animal protein, glycerin, dimethicone, simethicone, polyvinylpyrrolidone, propylene glycol, and stearalkonium chloride.[9] Protein-derived substances are popular conditioners for damaged hair, as they can temporarily mend split ends, also medically known as trichoptilosis. Split ends arise when the protective cuticle has been lost from the distal hair shaft and the exposed cortex splits. Protein is attracted to the keratin, a property known as substantivity, and the protein holds the cortex fragments together until the next shampooing occurs.[10]

## PH ADJUSTERS

Another way to minimize hair damage that may result from shampooing is to prevent the hair shaft from alkalinization. Most detergents have an alkaline pH, which causes hair shaft swelling. This swelling loosens the protective cuticle predisposing the hair shaft to damage. Hair shaft swelling can be prevented by ‘pH balancing’ the shampoo by the addition of an acidic substance, such as glycolic acid. Shampoos formulated at a neutral pH are most important for chemically treated hair, from either permanent dyeing or permanent waving.

## SPECIALTY ADDITIVES

The last and most important category of hair shampoo ingredients are the specialty ingredients. These additives allow the distinction of one shampoo from another in terms of marketing claims. The extra ingredients may provide unique functional attributes to the shampoo or may simply be added as the ingredient of the moment. A good example was the addition of beer, during the 1970s, to shampoos as an added conditioner. Presently, no beer containing shampoos are found in the marketing mainstream. During the 1990s, manufacturers were touting the addition of glycolic acid to shampoo. As mentioned previously, glycolic acid served functionally as a pH adjuster and nothing more. The current trend appears to be the addition of conditioning vitamins to shampoos, such as Vitamin B5 (panthenol). Other botanicals, such as tea tree oil, have also captured the interest of the moment. It is worth mentioning that shampoos are frequently reformulated to meet the marketing expectations of the consumer.

## SHAMPOO TYPES

There are many types of shampoos, too numerous to mention in this article, however, it is worthwhile discussing shampoos by basic type. The basic shampoo groups are listed in Table 3. Shampoos have been formulated in liquids, gels, creams, aerosols, and powders. Only the liquids will be discussed, as these are the most popular.

**Table 3:** Basic shampoo types

Normal hair shampoo
Dry hair shampoo
Damaged hair shampoo
Oily hair shampoo
Everyday shampoo
Deep cleaning shampoo
Baby shampoo
Medicated shampoo
Two-in-one shampoo
Hair dyeing shampoo

### Normal hair shampoo

Normal hair shampoos are designed to thoroughly cleanse the hair in persons with moderate sebum production who do not possess chemically processed hair. Mainly men fall into the category, although the fastest growing segment of hair dye sales are among young men! Normal hair shampoos use lauryl sulfate as the primary detergent providing good sebum removal and minimal conditioning.

### Dry hair and damaged hair shampoo

Dry hair shampoos provide mild cleansing and good conditioning. Many of the modern shampoos for dry hair are known as ‘2-in-1’ shampoos meaning that they contain a conditioner and a shampoo in the same product. It was the addition of silicone in the form of dimethicone, to these shampoos, that made this claim possible. Mild detergents, such as the laureth sulfates, are combined to give good foam production, but mild sebum removal. The detergents remove the environmental dirt and sebum from the hair in the water soluble phase, but the oil soluble silicone remains behind as a thin coating over the hair shaft. Thus, the product replaces sebum with silicone to make the hair shiny, soft, and free of static electricity. These products have revolutionized dry hair care.

### Oily hair shampoo

Oily hair shampoos are designed to optimize sebum removal from the scalp and hair shaft. This is accomplished by the selection of strong detergents and the minimal use of conditioners. The detergents employed are the lauryl sulfates or sulfosuccinates detergents. The use of conditioners on oily hair is not necessary due to ample sebum production.

### Everyday shampoo

A newcomer to the shampoo market is the everyday shampoo. Many persons feel that they do not have good hygiene unless they bathe daily. Technically, it is not necessary to shampoo the hair daily unless sebum production is high. Shampooing is actually more damaging to the hair shaft than beneficial. Everyday shampoos have been designed to meet the needs of the daily bather and generally contain mild detergents. They typically do not incorporate the conditioners found in the dry or damaged hair shampoos, and an instant conditioner can be used in combination with these products. Instant conditioners are applied immediately after shampooing in the shower and completely rinsed from the hair prior to drying.

### Deep cleaning shampoo

Deep cleaning shampoos, also known as clarifying shampoos, are at the other end of the spectrum from everyday shampoos. They are designed to thoroughly remove sebum from the hair shaft, but are typically used to remove retained styling products, such as hair spray, gel, and mousse. Everyday shampoos and dry hair shampoos are not good at removing the polymer film created by hair care products designed to keep the hair in place. These polymers can build up on the hair shaft after seven days use and leave the hair feeling harsh and appearing dull. In order to remove the polymer, a strong detergent in the form of a deep cleaning shampoo is used. These shampoos are typically used once weekly to keep the hair free of hair styling product build up. These shampoos, like the oily hair shampoos, use lauryl sulfates as their primary detergent.

### Baby shampoo

Baby shampoos are non-irritating to the eyes and designed as mild cleansing agents, as babies produce limited sebum. These shampoos use detergents from the amphoteric group, such as the betaines.[11] The detergent actually acts as an anesthetic and numbs the eye tissues to prevent stinging. Thus, eye damage can still occur if the baby shampoo is accidentally introduced into the eye, but the injury is not painful unless the anesthetic effect of the shampoo is no longer present.

### Medicated shampoo

Medicated shampoos are designed to deliver some other benefit to the scalp besides cleansing. Most medicated shampoos are aimed at relieving scalp itch and / or scaling. These products are classified as over-the-counter drugs, as they contain active agents such as, tar derivatives, salicylic acid, sulfur, selenium sulfide, polyvinylpyrrolidone-iodine complex, chlorinated phenols or zinc pyrithione.[12] Medicated shampoos have several functions: to remove sebum efficiently, to remove scalp scale, to decrease scalp scale production, and to act as an antibacterial / antifungal. The shampoo detergent removes the sebum, while mechanical scrubbing removes the scalp scale. Tar derivatives are commonly used as anti-inflammatory agents. Sulfur and zinc pyrithione are used for their antibacterial/antifungal qualities. Salicylic acid is added as a keratolytic and menthol is added to some shampoos to produce a tingling sensation that some patients find esthetically pleasing, and to provide a secondary stimulus, which neurologically decreases the perception of scalp itching.

### Professional shampoos

Professional shampoos are sold through beauty supply houses and are designed for special salon use. There are two types of professional shampoos: those intended for hair washing prior to cutting or styling and those intended to precede or follow a chemical process. Professional shampoos for hair washing are the same formulation as the over-the-counter varieties except that they are more concentrated and must be diluted eight to ten times before use. Otherwise, they contain the same formulation and variety of specialty additives discussed earlier. There is a perception among beauticians that salon shampoos are superior to mass market shampoos because they offer hair benefits not otherwise obtainable. It is hard to evaluate this opinion, as hair salons derive a significant profit from product sales. However, it can be stated that there are no ingredients present in salon shampoos that have not been incorporated into those sold in the general market.

A special subset of professional shampoos that are not sold to the general public or are sold only in conjunction with home hair dyeing products are the anionic, acidic professional shampoos used after bleaching to neutralize residual alkalinity and prepare the hair for subsequent dyeing. These shampoos perform a vital function in preventing irreversible hair damage during the bleaching procedure. When the hair is exposed to an alkaline pH, as discussed previously, the cuticular scale swells and makes the hair shaft more porous. The use of an acidic shampoo restores the normal cuticle configuration and prevents excessive dye damage to the hair shaft.

Another category of special professional shampoos are those designed for use after completion of the dyeing procedure. These are cationic, acidic shampoos used after dyeing to act as a neutralizing rinse. These products also decrease cuticular swelling to prevent the new color moieties from exiting the hair shaft, resulting in color fading. Subsets of these shampoos, known as ‘color revival shampoos’ in the mass market, are designed to maintain the color of bleached or dyed hair. These special shampoos designed for use with hair coloring procedures are generally only available to licensed cosmetologists, as they are not appropriate for daily cleansing.

## ADVERSE REACTIONS

Adverse reactions to shampoos are rare, as the product is rinsed away from the skin quickly allowing insufficient time for the development of allergic or irritant contact dermatitis. Probably the most common problem with shampoo is accidental contact with the mucous membranes, such as the nose and eye. The possible causes of allergic contact dermatitis are listed in Table 4.[13]

**Table 4:** Causes of shampoo-induced allergic contact dermatitis

Formalin
Parabens
Hexachlorophene
Miranols

If patch testing to a shampoo is required, the shampoos should be diluted to form a 1 – 2% aqueous solution for closed patch testing and a 5% aqueous solution for open patch testing. However, it should be recognized that false positive reactions due to irritation may still occur. A better assessment may be obtained by patch testing individual ingredients separately.[14]

## SUMMARY

Shampoos are a complex formulation of ingredients selected to clean the scalp, as well as beautify the hair. This is a complex task leading to the wide variety of shampoos currently in the market. Selecting the proper shampoo for a patient with a given dermatologic hair disorder means understanding the various ingredient functions and the unique attributes of each shampoo type. This article has presented some fundamental knowledge in this regard.
